# Design of a Non-Contact Radial Torque Sensor with Variable Torque Range by Electromagnetic Coil Coupling with Piezoelectric Sensor

**DOI:** 10.3390/ma14247695

**Published:** 2021-12-13

**Authors:** Sheng-He Wang

**Affiliations:** Department of Mechanical Engineering, Southern Taiwan University of Science and Technology, Tainan 71005, Taiwan; sunhur0315@stust.edu.tw; Tel.: +886-6-2533131 (ext. 3528)

**Keywords:** contactless torque sensor, piezoelectric-loading sensor, electromagnetic coupling force, static force measurement

## Abstract

Recently, due to the development of automation technology, torque measuring and monitoring technologies have been brought to the focus. However, the commercially available sensors have the disadvantage of large volume, which results in the difficulty of installation on existing automated machines. Responding to the above-mentioned problem, a contactless torque sensor that uses an electromagnetic coil combined with a permanent magnet was proposed. By adjusting the input electric current in the coil, the strength of the magnetic field can be controlled to generate a non-contact magnetic force to resist external torque loading. For the measurement of such a magnetic force, a cantilever-beam mechanism comprising a piezoelectric-loading (PZT-L) sensor is employed to estimate the external static force by measuring the variation of the electric impedance. According to the measured results, the proposed PZT-L sensor demonstrates the accuracy of the proposed design, for which the maximum estimated error was around 6%. Finally, the proposed contactless torque sensor with 11 cm in diameter and 2 cm in thickness was employed to verify the effectiveness of theoretical analysis. From the sensor characteristic measurement, the detection range for external torque can be from 7.8 to 125.6 N-mm when the driven current input ranged from 2 to 10 A. Therefore, the experimental results presented that the moment of inertia via the resisted torque can be adjusted by the proposed non-contact torque-sensing system according to the measuring condition.

## 1. Introduction

**Figure 1:**
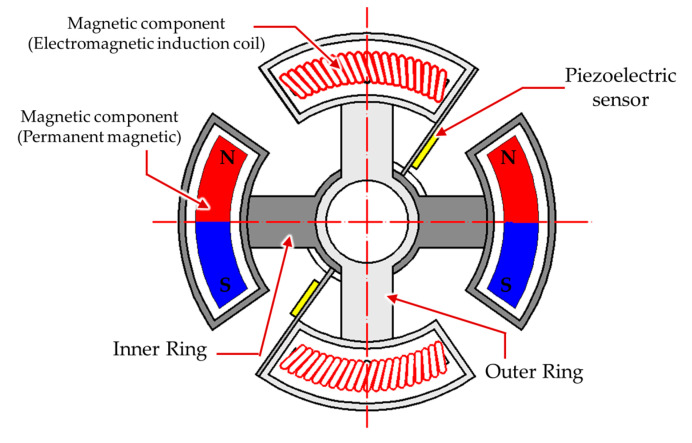
The structure of the proposed contactless radial torque sensor.

Recently, to reduce production costs and manpower resources, intelligent automated manufacturing has been actively developed. In the development of automation technology, safety and reliability have always been the key issues to inspire the investing intention of manufacturers. To enable a robot/robotic arm to safely and reliably execute various command actions, the operating information, such as the position, velocity, acceleration, force, temperature, etc., must be obtained and monitored. However, the force or torque sensitivity is difficult to detect for humans, which can easily result in harm. Moreover, considering that an electric motor is usually utilized as the power source of automation equipment or robotic arms, motor-torque measurement is the most important consideration for safety-monitoring applications. In a traditional rotating-torque sensor, two electric coils are placed oppositely in the radial direction, which induces an electric current when two of the electrical coils have an angle offset by the external toque loading [1]. Inducing a sufficient output voltage from the electrical coil is difficult, and commercial rotating torque sensors have the disadvantage of large dimension in the axial direction; as such, assembly into available production equipment is problematic. Therefore, the development of a thin-dimension torque sensor is necessary. Regarding the generally driven principle of the torque sensor, after the external torque load is applied in the internal shaft in the sensor, a torsion angle will be generated. Then, the measured torsion angle information of the internal shaft can be utilized to estimate the external torque through the material mechanics. To measure the torsion angle, an intuitive approach is to adhere the strain gauge on the deforming shaft, from which the voltage variation can be measured to calculate the torsion angle of the shaft [2]. However, the torsion angle of the shaft must be sufficiently large for the strain gauge, which leads to large dimensions and measuring inaccuracy. To detect the tiny torsion deformation, some contactless measuring technologies have been developed and investigated, and they can generally be separated into the optical, electromagnetic induction, and piezo-inductance methods. In 1996, it was proposed that the two ends of an internal shaft use a magnetically conductive metal. When a twist torsion occurs in the shaft by an external torque, the expansion or contraction deformation at the two ends will change the permeability of the magnetically conductive material. Then, the degree of torsion can be estimated to obtain the external torque by measuring the permeability of the two ends [3]. Another method of measuring the twist torsion of the shaft by using the induction coil, for example, arranged magnets in parallel with a 45° and 0° in the middle of a shaft, was proposed [4,5]. When an external torsion load is applied, the induced voltage from the external coil could be detected by the arrangement magnets moving, and the twist torsion and torque loading can be estimated. Since there is a time delay with the electromagnetic induction method, this approach is not suitable for application in high-frequency torsional loadings. Therefore, a plane-type, fast-response piezoelectric sensor was proposed to replace the strain gauge [6]. In this manner, a voltage generated through the piezoelectric effect can be used to detect the high-frequency torsional load. Similarly, by using the piezoelectric element, a surface acoustic wave can be generated, which flows across the surface of the shaft. During the torsional deformation process, the rigidity of the rotating shaft will vary, resulting in wavelength changes of the surface acoustic wave, which can be utilized to estimate the torsional deformation information [7]. However, a piezoelectric element operating at high frequency is easily affected by environmental factors, while fluctuations in the generating voltage can cause measuring inaccuracies. Moreover, these non-contact torque measurement technologies were all proposed to detect the torsional deformation of an internal shaft. Therefore, to improve the measuring accuracy and sensitivity, the length of the shaft should be increased to obtain sufficient torsion deformation, and the restriction of the shaft material is not suitable for measuring small torque loads [8]. In this paper, a contactless torque sensor that uses magnetic force is proposed, as shown in Figure 1.

The proposed contactless torque sensor is designed to assemble inner and outer rings together by a ball bearing to form a thin roundel structure, and the magnetic components are arranged radially. To achieve the variable range of the torque measurement, an electromagnetic induction coil is proposed instead of one side of the magnetic poles. To improve the wiring issue and generate a contactless magnetic force, when the inner ring with the helical induction coil is fixed, the external torque load is applied on the outer ring to bring on the rotation, and it can be resisted by the repulsive force depending on the interval distance of the magnets, for which the relationship is similar to the spring force. To obtain the magnetic repulsive force, considering the dimension limitation and the narrow output voltage range for a low measuring resolution, in this paper, a thin piezoelectric plate of a force sensor, in which one side of the piezoelectric unimorph plate is mounted in the center of the proposed roundel sensor to be a cantilever structure, is designed to measure the contactless magnetic force for its advantages of a compact size and simple construction. Regarding the other side of the piezoelectric cantilever sensor fixed on the rotated outer ring, a bending deformation will occur during two magnetic fields getting close, which is utilized to estimate the loading force by measuring the electric impedance variation according to the fast electromechanical conversion characteristics. Finally, because the coupling magnetic field can be controlled by the electric current of the induction coil and interval distance between two of the magnetic poles, the designed torque sensor demonstrates the dynamic variable characteristics in the moment of inertia that the resisted torque can be adjusted according to the measuring condition.

## 2. Theoretical Analysis of Electromagnetic Force

In this paper, a contactless magnetic force was utilized to measure the external torque load. From the basic law of magnetism [9], a repulsive force from similar magnetic poles can be generated to resist the external torque loading. Then, the shorter interval distance between two of the magnetic poles can be excited to produce a higher repulsive force, the relationship of which is similar to the spring force. To analyze the repulsive force, the relation of the magnetic field strength can be calculated by [10]

(1) $$F=\frac{1}{4\pi \mu_{0}\mu_{r}}\frac{\phi_{1}\phi_{2}}{d^{2}},$$

where $\mu_{0}$ is the vacuum permeability of the free space, the value of which is generally 1.25663706212×10−6 (NA2) [11]; and $\phi_{1}$ and $\phi_{2}$ are magnetic fluxes of two magnets, respectively. Observing Equation (1), the repulsive force will be affected by the square of the distance *d* between two magnets, which means that the lager repulsive force can be found in the shorter distance of the two magnets.

Moreover, to achieve the variable range of the torque measurement, an electromagnetic induction coil was proposed instead of one side of the magnetic poles in this paper. According to the Ampere screw rule [12], when an electric current flows through a helical coil, an electromagnetic field can be induced. To improve the electromagnetic strength, a round bar-shaped iron core with permeable magnetic material is located in the center of the helical coil to converge the induced magnetic field. Therefore, by using Ampere’s original law and Biot–Savart’s law [12], depending on the electric current in the helical coil *I*, the magnetic flux density of the electromagnetic induction coil can be represented as:

(2) $$\oint^{\;}\vec{B}\cdot d\vec{l}=\mu_{0}\mu_{r}\iint_{s}^{}\vec{J}\cdot d\vec{s},$$

where $\vec{B}$ is the magnetic flux density parallel to an infinitesimal vector length $d\vec{l}$, and $\mu_{r}$ is the relative magnetic permeability of an iron core in an electromagnetic coil. $\vec{J}$ is the current density (Am2), which is normal to an infinitesimal vector area of the surface $\vec{s}$. $\vec{B}$ and $\vec{J}$ are both the vector quantities, and the orientation of $\vec{B}$ related to $\vec{J}$ can be obtained by the right-hand rule. Therefore, when considering a finite length and homogeneous resistance of a solenoidal coil [13] for an enclosed path, the flux density within the coil can be rewritten as [13]:

(3) $$B_{C}=\frac{\mu_{0}\mu_{r}N_{C}I_{c}}{L_{p}},$$

where $L_{p}$ is the length of the iron core, and $N_{C}$ is the number of coil turns, where the electric current in each turn is presented as $I_{c}$. By observing Equation (3), the intensity of the magnetic flux generated by the electromagnetic coil is proportional to the number of turns and the electric current in the wire, but it is reduced in accordance with the length of the iron core due to the average magnetic path being extended. Furthermore, the direction of flux density is also parallel to the lengthwise dimension based on the right-hand rule. Finally, according to Equation (1), the magnetic flux of the electromagnet coil can be estimated by the induced flux density from Equations (2) and (3), and it is represented as:

(4) $$\phi_{1}=\int^{\;}\vec{B}\cdot d\vec{A},$$

where *A* is the surface with unit area, which should be perpendicular to the magnetic flux to produce the magnetic field. Since the magnetic flux is just a way of expressing the magnetic field in a given area, its SI unit of magnetic flux is defined via Weber (Wb). Therefore, from Equations (1) to (4), the input current in the electromagnet coil can affect the magnetic field strength; in addition, the repulsion force is induced by controlling the interval distance between two of the magnetic fields.

**Figure 2:**
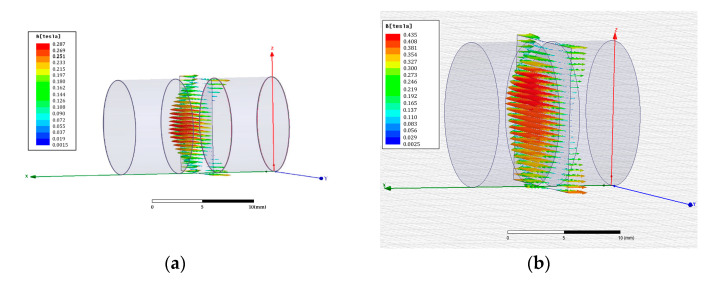
Simulation results for the plane distribution of the magnetic field in the center of the spacing between two equivalent magnetic pieces by finite element analysis software; (**a**) 5 mm interval distance; (**b**) 3 mm interval distance.

To observe the magnetic field strength related to the interval distance, Ansoft, a finite element analysis software was employed to simulate the strength of two magnetic fields coupling with different interval distances. To simplify the simulated process, based on Equation (3), if a 100 A input electric current is assumed in a designed electromagnetic coil, its flux density can be estimated to be around 0.63 (Tesla) by its specifications, as shown in Table 1. Then, in the simulation process, the designed electromagnetic coil is simplified as an equivalent magnetic piece with the calculated flux density, the dimensions of which are defined as 12 mm in diameter and 6 mm in thickness, which is the same size as the permanent magnet (NdFeB). Finally, according to the specifications of the permanent magnet, as shown in Table 1, the variation of the magnetic field intensity can be simulated when the equivalent magnetic piece is close to the opposing permanent magnet. By the finite element analysis software, the intensity of the magnetic field between two equivalent magnetic pieces can be simulated, and the plane distribution in the center of the spacing with different interval distances, namely 5 mm and 3 mm, is shown in Figure 2. 

**Figure 3:**
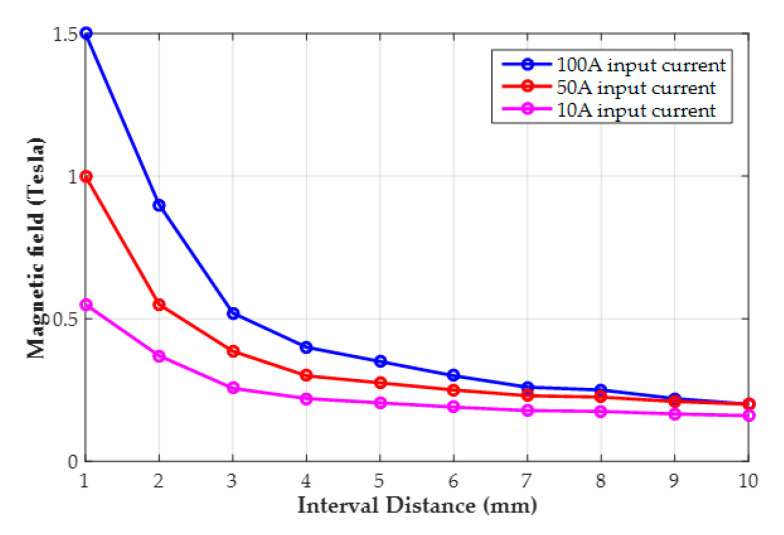
Simulation results for the maximum magnitude of the magnetic field in the center of the spacing with different interval distances.

Compared with the simulated results from the 5 mm and 3 mm distances, it can be found that when two magnets are moved close to each other, the magnetic flux line will converge to increase the strength and range area of the magnetic field, respectively. Furthermore, based on the induced magnetic flux with different input currents from Equation (3), the simulated results of the maximum magnetic field magnitude in the center of the spacing with the interval distance being 10 to 1 mm can be recorded and demonstrated, as shown in Figure 3. Observing Figure 3, with the same induced magnetic flux of the input current, the strength of the converged magnetic flux will be increased sharply when the equivalent induced-magnetic is close to the permanent magnet, for which the description of the curve relation corresponds to Equation (1). Moreover, depending on increasing the input current of the electromagnetic coil, the high induced-magnetic flux can be generated to significantly improve the coupling field intensity. However, the induced magnetic field strength from the coil is not strong enough to quickly attenuate the coupling magnetic flux as the permanent magnet moves away, and a similar range of intensity can be found when the interval distance is over 10 mm.

**Figure 4:**
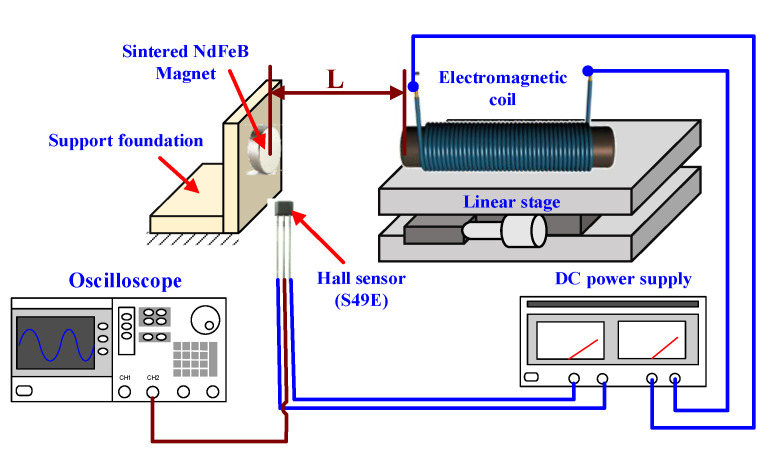
The experimental scheme of the magnetic flux strength by electromagnetic coil coupling with a permanent magnet.

To confirm the theoretical analysis, an experimental measurement scheme is proposed, as presented in Figure 4. The testing electromagnetic coil is fixed on a linear stage to adjust the interval distance with the opposing side of the permanent magnet, the material of which was chosen as a sintered type of NdFeB, and it is fixed on the support foundation. To measure the magnetic field intensity, a hall sensor (Model: S49E) is proposed and placed around the edge of the permanent magnet surface. From the working principle of the hall sensor, a DC voltage should be used as the drive voltage, the charge carriers of which are deflected by the external magnetic field to produce a difference in electric potential (voltage). Therefore, a DC power supply is utilized as the drive voltage of the hall sensor and the input current of the testing electromagnetic coil. Finally, the output voltage of the hall sensor is recorded and represented on an oscilloscope, and the change of the magnetic field strength is measured.

**Figure 5:**
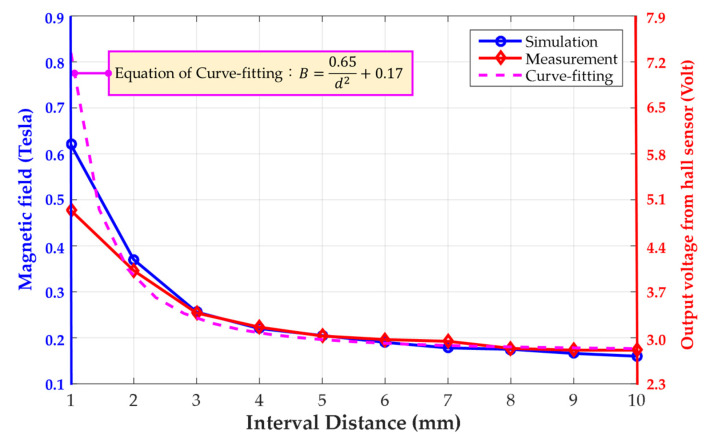
Curve fitting for the simulation result with a 10 A input current and compared with the output voltage from the hall sensor.

According to the proposed experimental measurement scheme, when the testing electromagnetic coil with a 10 A input current is moved close to the permanent magnet, the output voltage from the hall sensor is detected and compared with the simulated results of the magnetic field intensity. As the interval distance changes from 10 to 1 mm with a pitch of 1 mm, Figure 5 shows the comparison results, for which the left-hand scale of the chart represents the magnitude of the magnetic field in simulation, while the right-hand scale is the output voltage measured from the hall sensor. It can be seen that the curvilinear trend of the coupling field related with the interval distance from the simulation is similar to the voltage of the hall sensor, of which the intensity is demonstrated as gradual increments as the two magnetic fields approach. Therefore, based on the curve-fitting method, the relationship between the magnetic field and interval distance can be obtained, as shown by the dashed line in Figure 5,

(5) $$B=\frac{0.65}{d^{2}}+0.17,$$

where *d* represents the interval distance between the coil and permanent magnet. 

Observing Equation (5), the hall sensor is designed to be located around the edge of the permanent magnet surface, and a magnetic remanence of 0.17 T from the permanent magnet without the magnetic coupling effect can be found when the interval distance is over 10 mm. In addition to the magnetic remanence, the coupling intensity of the magnetic field related to the interval distance by the curve-fitting method is represented as an inverse function of the square of the interval distance, which is similar to the repulsive force of Equation (1). Furthermore, based on the specification of the chosen hall sensor, the range of the output voltage is significantly limited from 2.5 V and 5 V for the positive magnetic field detection, of which the narrow output range with the proportional relation results in low measuring resolution when used in switching applications in particular. Therefore, in this paper, a piezoelectric sensor is designed instead of the hall sensor to utilize the repulsive force measurement during the closing of two magnetic fields.

## 3. Design and Theoretical Analysis of Piezoelectric Sensor

**Figure 6:**
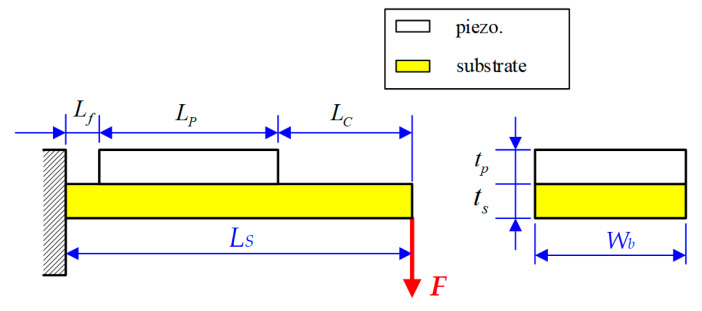
Designed dimensions of the proposed unimorph-type cantilever structure.

In our designed torque-sensor structure, the contactless magnetic force can be adjusted to resist the variable range of the torque loading by the coil input current. However, the magnetic coupling intensity is difficult to measure using the low measuring resolution of the hall sensor. Therefore, in this paper, the piezoelectric material was employed to transfer the deformed displacement into electric energy by its electromechanical converting properties. Furthermore, considering that piezoelectric material also offers other advantages, such as compact size, simple construction, and the capability of high electromechanical conversion efficiency [15], a piezoelectric sensor was designed and developed to detect the repulsive force from the magnetic coupling field with similar poles; as such, the external torque loading can be further estimated. To provide superior detecting sensitivity, a cantilever structure for the piezoelectric sensor was proposed for its high deformation behavior in a limited force application, as shown in Figure 6. Observing Figure 6, the proposed piezoelectric sensor constitutes a unimorph form of a two-layer bender for which the structure is coupled to a piezoelectric plate fixed to a steel plate substrate. To obtain higher deformation during the force application, the piezoelectric plate should be located around the middle of the bender such that one side of the steel substrate is mounted in the fixed support to be a cantilever structure.

**Figure 7:**
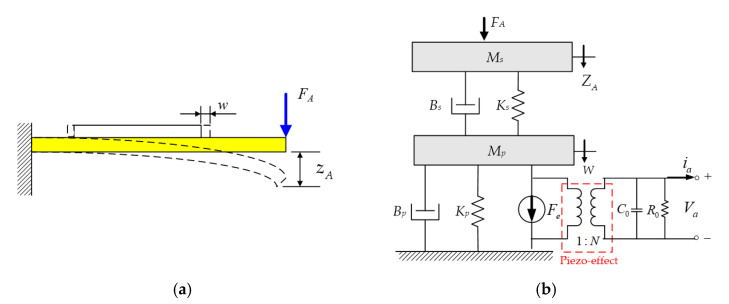
The deformation situation of the unimorph cantilever bender by the external force; (**a**) schematic diagram of loading and deformation for the unimorph cantilever; (**b**) equivalent electromechanical model to describe the relationship between the external force and deformed displacement [16].

To analyze the electric output response of the piezoelectric sensor under the different mechanical input conditions, the deformed displacement of the unimorph-type cantilever beam is shown in Figure 7a. It can be found that an external force $F_{A}$ applied on the front (free side) of the unimorph bender results in bending deformation, which is defined as $z_{A}$. Then, the fixed piezoelectric plate is stretched to generate an internal extending stress inside, which also results in transverse deformed displacement as w to convert into electrical energy output. 

To build the theoretical electromechanical model of the piezoelectric sensor, following the author’s previous paper [17], the electromechanical converting relationship can be represented by the piezoelectric constitutive equation in the strain-charge form with d-type, as follows [18]

(6) $$S_{1}=s_{11}^{E}T_{1}+d_{13}E_{3},$$

(7) $$D_{3}=d_{31}T_{1}+\varepsilon_{33}^{T}E_{3},$$

where $T_{1}$ and $E_{3}$ are the mechanical stress and electric field of the system input, and they result in the mechanical and electrical generation of strain $S_{1}$ and electric displacement $D_{3}$, respectively. The independent in–out relation is defined as the mechanical elastic compliance $s_{11}^{E}$ and electric permittivity coefficient $\varepsilon_{33}^{T}$ under constant strain. Moreover, a piezoelectric coefficient *d*_31_ can be found to represent the electric–mechanic conversion relation. Finally, the subscripts of the variables are the respective in–out applied direction, where the horizontal and polarized directions are denoted as 1 and 3, respectively. In order to describe the electromechanical coupling relation, according to the author’s previous paper, an equivalent mass-spring-damper (MSD) model with a single degree of freedom [18] is proposed, as shown in Figure 7b. Regarding the unimorph-type cantilever structure of the proposed piezoelectric sensor, coupling two of the equivalent mass-spring-damper (MSD) models was used to represent each of the mechanical properties. When an external force $F_{A}$ is applied on the steel substrate of the unimorph bender, the equivalent mechanical model can be represented as an equivalent mass $M_{s}$ connected in parallel with a spring $K_{s}$ and a viscous damper $B_{s}$, and then, the stretched piezoelectric plate also behaved as another MSD model of $M_{p}$, $B_{p}$, and $K_{p}$. From the cantilever deformed schematic in Figure 7b, each displacement $z_{A}$ and w is defined as the bending and extending amplitudes, respectively. Furthermore, in order to describe the electric–mechanic conversion characteristic, based on the relation with the electric displacement field, the constitutive equations of Equation (7) can be rewritten by Gauss’s law, as follows [19]:

(8) $$Q_{3}=\iint^{\;}D_{3}dA_{s}=\iint^{\;}d_{31}T_{1}dA_{s}+\iint^{\;}\varepsilon_{33}^{T}E_{3}dA_{s},$$

where $Q_{3}$ is defined as the electric charge on the surface of the piezoelectric material with the polarized direction, while $A_{s}$ is denoted as the cross-section area in the horizontal direction. Assuming that a uniformly electric field is distributed on the piezo-ceramic plate, then all of the physical relations can be simplified as [20,21]:

(9) D3=Q3As, E3=Vet, T1=FtAs,

where *t* is the thickness of the piezo-ceramic plate and $V_{e}$ is its electric field distributed on the surface. Furthermore, a mechanical shear stress $T_{1}$ can produce an external force with a horizontal direction, which is denoted as $F_{t}$, and also convert it into the electric charge $Q_{3}$. Therefore, according to the relationship in (9), the constitutive equations of the electromechanical coupling relation for the piezoelectric sensor can be rewritten as:

(10) $$Q=N\cdot F_{t}+C_{0}V_{e},$$

where N is the electromechanical converted coefficient ($N=\frac{d_{31}A_{s}}{t}$). Furthermore, it can be found that the piezoelectric material has a capacitor characteristic, for which the coefficient is denoted as $C_{0}=\frac{\varepsilon_{33}^{T}A_{s}}{t}$. Therefore, to represent the electric property, an equivalent circuit, for which a resistor $R_{0}$*_,_* is connected to a capacitor $C_{0}$ in parallel, is given to couple with the MSD model, as shown in Figure 7b, and a transformer circuit is used to describe the electric–mechanic conversion relation with the converted ratio as N. 

**Figure 8:**
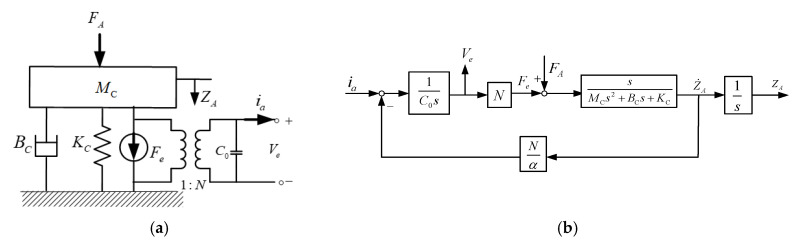
To represent the overall input–output relation of the PZT sensor, the proposed theoretical models are built; (**a**) a single MSD model coupled with the equivalent circuit; (**b**) a system block diagram with the Laplace transformation.

From Figure 7b, it can be found that the equivalent circuit also represents the electric loss property for the piezoelectric sensor, and the equivalent resistor *R*_0_ is often, in practice, large enough to ignore the electric loss in resonance frequency operation [22]. Moreover, to simplify the mechanical dynamic response, consider that the small bending deformation and the extending deformed displacement of the piezoelectric plate w are linearly related with the front of the unimorph bender and can be represented as $z_{A}=\alpha \cdot w$. Then, the dynamic model of Figure 7b can be simplified to a single MSD model, as shown in Figure 8a, for which the converted relation can be given by

(11) $$\mathrm{The}\;\mathrm{equivalent}\;\mathrm{system}\;\mathrm{mass}:\;M_{C}=M_{s}+\frac{M_{p}}{\alpha },\;\mathrm{The}\;\mathrm{equivalent}\;\mathrm{system}\;\mathrm{damping}:\;B_{C}=B_{s}+\frac{B_{p}}{\alpha },\;\mathrm{The}\;\mathrm{equivalent}\;\mathrm{system}\;\mathrm{spring}:\;K_{C}=\frac{K_{s}+M_{p}}{\alpha }.$$

Therefore, to analyze the electric output related with external force, based on the proposed electromechanical model in Figure 8a, a feedback block diagram with the Laplace transformation to represent the overall input–output behavior of the piezoelectric sensor is given by Figure 8b. Regarding the electromechanical characteristic of the piezoelectric material, two of inputs and outputs can be found depending on its application, which are the electric current $i_{a}$ and the front bending displacement $z_{A}$ as the system input and output for application in the actuator practice, respectively. Similarly, in our sensor application, the system input utilizes the external force $F_{A}$, and so, the electric voltage $V_{e}$ will become the output response. In addition to analyzing the output response of the designed piezoelectric sensor, based on the proposed feedback block diagram model of Figure 8a, the transfer function of the output voltage related with the external force can be estimated by Mason’s rule [23].

**Figure 9:**
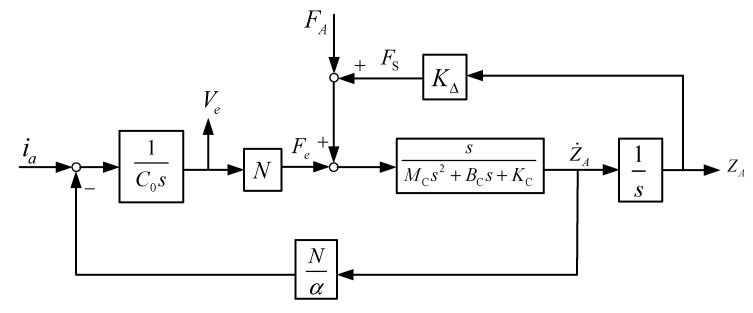
The system block diagram with a steady ambient force $F_{S}$.

However, in observing the theoretical electromechanical model, it can be found that the piezoelectric material has an inherent electric-loss property from the internal capacitance and resistance characteristics, which gradually reduces the generating voltage by external force over time. Therefore, for the fast electromechanical conversion characteristic, a piezoelectric sensor is appropriate, particularly for ultrasonic-frequency measurement. Nevertheless, in this paper, the designed piezoelectric sensor is utilized to measure the coupling magnetic force with steady state. To achieve the steady force measurement, based on the MSD model from Figure 8a, an ambient force $F_{S}$ can be equivalent to the mechanical resistance representation, as given by

(12) $$F_{S}=M_{C}\ddot{Z_{S}}+B_{C}\dot{Z_{S}}+K_{C}Z_{S},$$

where $Z_{S}$ is the deformed displacement during the ambient force $F_{S}$ application. Therefore, assuming the ambient force has a stable loading on the piezoelectric sensor without vibration, then the transient response of Equation (12) for the vibration velocity and acceleration factor can be equal to zero ($\ddot{Z_{S}}=0,\;\dot{\;Z_{S}}=0$). Regarding the original system output in Figure 8b, it is assumed that $Z_{S}$ relating with $Z_{A}$ is an amplified relationship represented as $Z_{S}=\beta \cdot Z_{A}$, where β is denoted as the amplified factor depending on the ambient force $\left(\beta =\beta \left(F_{S}\right)\right)$; then, the ambient force $F_{S}$ can be rewritten as

(13) $$F_{S}=K_{C}Z_{S}=K_{\Delta }Z_{A},$$

where $K_{\Delta }$ is the equivalent spring coefficient when the deformed displacement with the ambient force is equal to the system output $Z_{A}$, while the converted relation is represented as $K_{\Delta }=\beta \left(F\right)\cdot K_{C}$. Therefore, Equation (13) can be combined with the system block diagram in Figure 8b and represented as shown in Figure 9. It can be found that the bending deformation by an ambient force will result in variations in structure resistance; in particular, the mechanical impedance can be utilized for steady external loading detection. However, the mechanical impedance comprises the ratio of the applied force related with the resulting velocity, and it is difficult to measure. Therefore, according to the electromechanical converted characteristic of piezoelectric material, the electric impedance, instead of the mechanical resistance, is proposed for detecting the steady external force in this paper. Based on the system block diagram of Figure 9, consider an ambient force $F_{S}$ only applied to the piezoelectric sensor without other external forces ($F_{A}=0$); in such a case, the electric impedance with Laplace formula from $i_{a}$ to $V_{e}$ can be calculated by Mason’s rule, and it is given as

(14) $$Z_{e}=\frac{V_{e}}{i_{a}}=\frac{M_{C}s^{2}+B_{C}s+K_{C}+K_{\Delta }}{C_{0}s\left(M_{C}s^{2}+B_{C}s+K_{C}^{*}+K_{\Delta }C_{0}\right)},$$

where $K_{C}^{*}=\frac{N^{2}}{\alpha B7C_{0}}$ is the equivalent feedback spring coefficient from electricity. From the basic mechanical MCS system, it is well known that the system resonance frequency $\omega_{n}$ can be affected by its mass and spring coefficient ($\omega_{n}=\sqrt{\frac{K}{M}}$). Similarly, to analyze the variation of electric impedance, based on the system theoretical model of Figure 9, the electrical resonance frequency $\omega_{en}$ can be estimated from Equation (14), and it is given as

(15) $$\omega_{en}=\sqrt{\frac{K_{C}^{*}+K_{\Delta }C_{0}}{M_{C}}}.$$

It can be found that the deformed displacement generated by an ambient force $F_{S}$ results in variations in structure resistance ($K_{C}^{*}+K_{\Delta }C_{0}$), and so, the resonance frequency at the lowest electric impedance can be changed and is utilized to estimate the external steady force.

**Figure 10:**
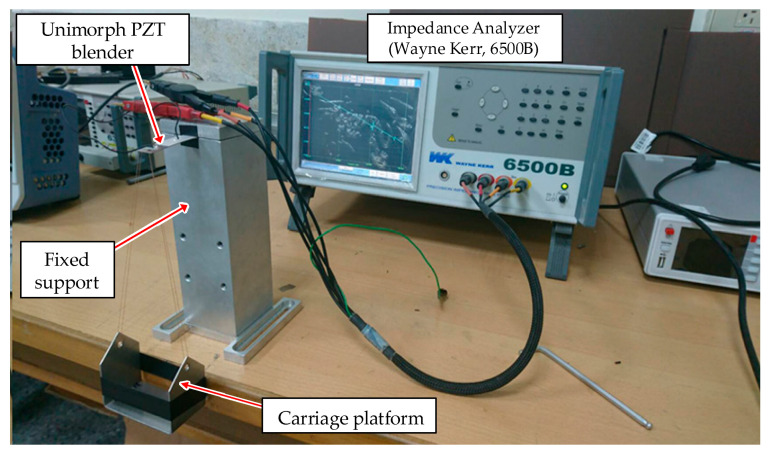
Stable loading-force experiment for the cantilever type of piezoelectric sensor.

To verify the theoretical analysis, a measuring structure with a stable loading force for the cantilever-type piezoelectric sensor was assembled, as shown in Figure 10, according to the dimensions of a unimorph bender with the piezoelectric and steel plate listed in Table 2, which was utilized in the author’s previous harvesting application [24]. A unimorph form of a two-layer bender can be constructed, one side of which is mounted in the fixed support to act as a cantilever structure. Moreover, a carriage platform is utilized to connect the free side of the unimorph bender, with an equivalent standard weight placed to simulate a stable loading force. To analyze the variation of electric impedance, an impedance analyzer (Wayne Kerr, 6500 B) is used to measure the electric impedance distribution under different loading forces.

**Figure 11:**
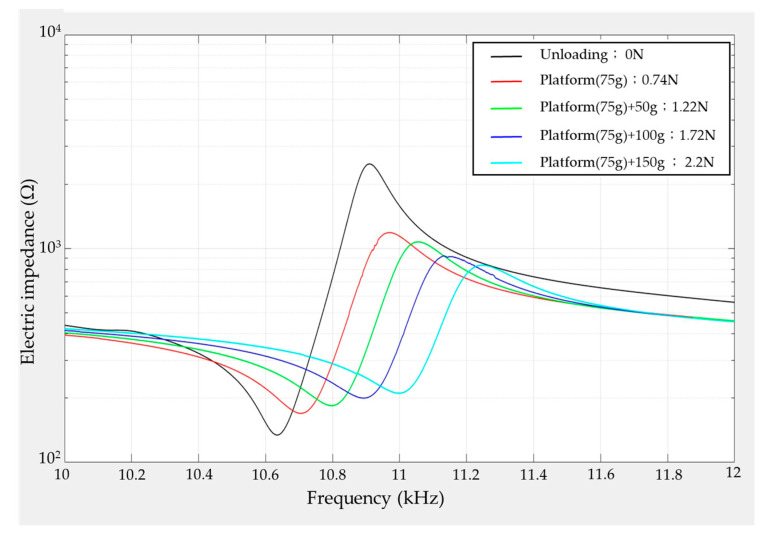
Experiment results of the electric impedance distribution with frequency under the different loading forces.

Figure 11 shows the measuring results of electric impedance under different stable loading forces based on the experiment scheme of Figure 10. Observing the electric impedance distribution, a tendency of the impedance to suddenly drop and then jump to a higher impedance value can be seen, where the frequency at the lowest and highest magnitudes in this limited range is defined as the resonance and anti-resonance frequencies, respectively. Accordingly, the resonance frequency can be found at around 10.63 kHz from the measured data. Moreover, it can be observed from the measured data that the electric impedance of the cantilever piezoelectric sensor is affected by the external loading force. 

**Figure 12:**
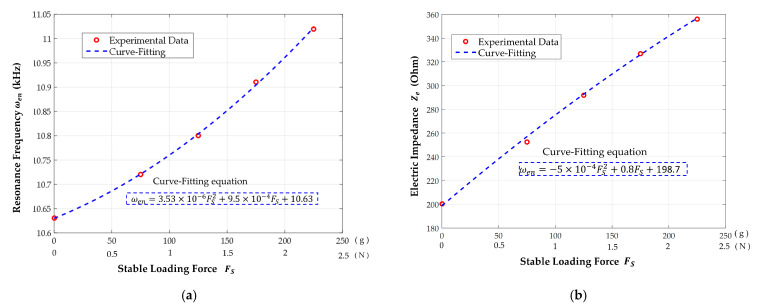
To observe the influence of loading force, the resonance frequency and electric impedance variation are used to analyze (**a**) the shift of resonance frequency and (**b**) the variation of electric impedance at the 10.55 kHz operation frequency.

To analyze the influence of the loading force, the resonance frequency and electric impedance variation with different loading weights was recorded, as shown in Figure 12. According to the shifting record of the resonance frequency in Figure 12a, it can be seen that the resonance frequency becomes increasingly dependent on the heavier loading, for which the increasing range is around 10.63 to 11.03 kHz from an unloaded state to a 225 g loading weight. Moreover, the increasing tendency is equivalent to a quadratic polynomial equation by the curve-fitting method, as represented by the equation in Figure 12a, and it can be used to estimate the external loading force. However, in the practical force measuring application, it is difficult to obtain the shifting measurement of the resonance frequency in real time. Therefore, operating at the same frequency as the drive voltage is proposed, and its corresponding impedance value is represented in Figure 12b for a 10.55 kHz operation frequency in accordance with the measured data of Figure 11. This demonstrates that the impedance value will increase during heavier loading applications, and this increasing relationship can be similarly calculated by the curve-fitting method. Therefore, in this paper, the same frequency of the drive voltage, namely 10.55 kHz, is chosen to operate the cantilever piezoelectric sensor; then, its electric current is utilized to estimate the external loading force with respect to the variation of electric impedance.

## 4. Measurement and Achievement of the Designed Contactless Torque Sensor

**Figure 13:**
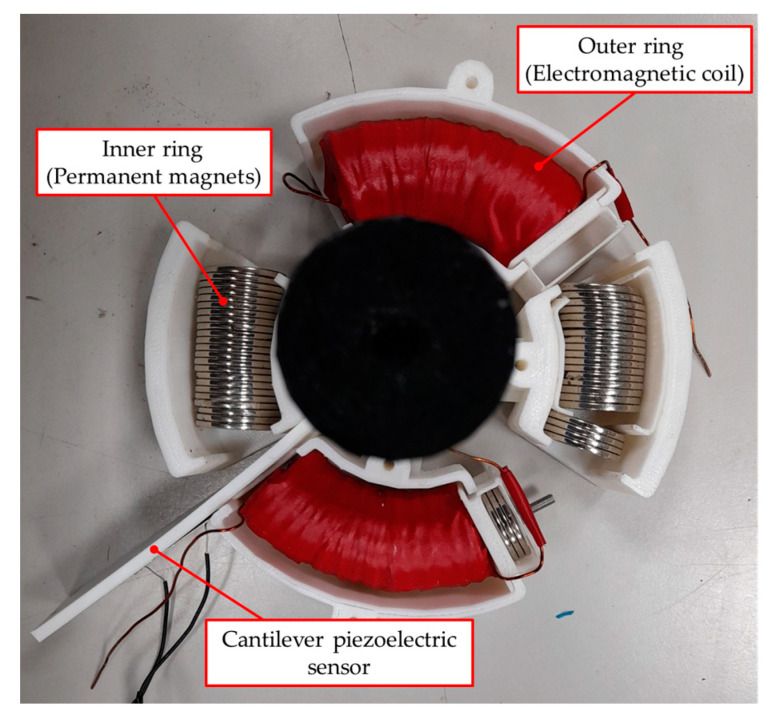
Picture of the proposed thin-dimension contactless torque sensor.

According to the aforementioned theoretical analysis, two couples of an electromagnetic induction coil with permanent magnet were combined to be a thin dimension of contactless torque sensor, as shown in Figure 13, the dimensions and material parameters of which are presented in Table 3. The appearance of the designed contactless torque sensor is a thin roundel structure with a diameter and thickness of approximately 110 mm and 20 mm, respectively, and the structure can be separated into two parts: one inner ring component in which the designed electromagnetic coil is located, and one outer ring component assembled in the stack of the permanent magnets. From the scheme of the designed contactless torque sensor, the interval gap between the inner and outer ring with a magnetic component was designed to be around 12°, which was converted around 8 mm space distance. Moreover, to improve the wiring issue and magnetic coupling strength, the inner ring is fixed, and some magnets are stacked on the front of the coil. Therefore, external torque applied on the outer ring is resisted by the repulsive force from the similar poles of the magnetic coupling field, and the repulsive force can be controlled by the interval distance between two of the magnetic poles and the electric current in the coil.

**Figure 14:**
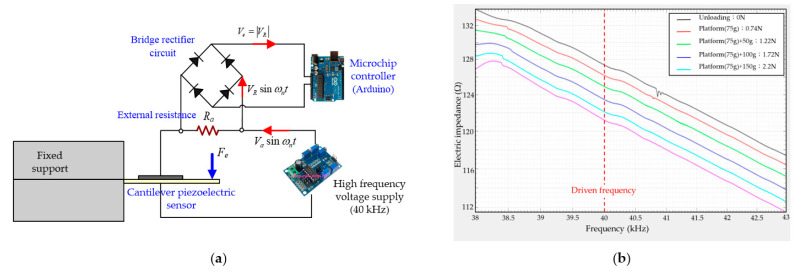
Measuring structure of high-frequency electric current for the cantilever piezoelectric sensor: (**a**) The measuring scheme from high frequency to DC voltage; and (**b**) based on the chosen drive circuit, the electric impedance distribution with different loading weights around 40 kHz.

**Figure 15:**
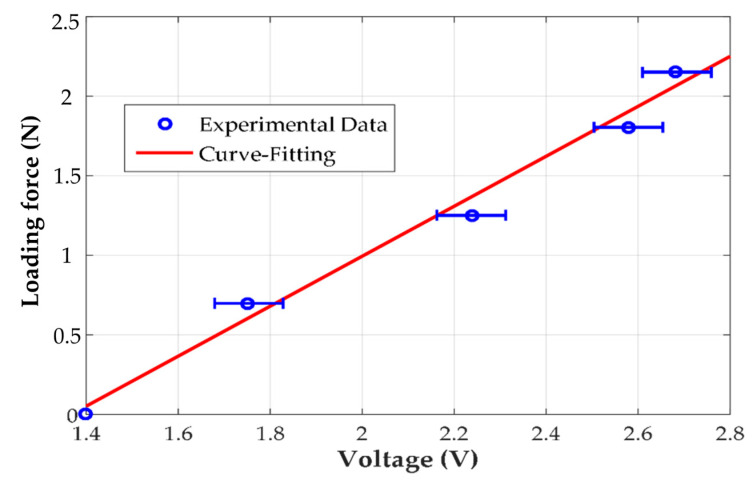
The measured data of the voltage drop across the external resistance related with different external loading forces.

In this paper, the impedance variation of the cantilever piezoelectric sensor is used to estimate the external loading force. However, in order to clearly observe the impedance variation, a high-frequency voltage source approximated to the system resonance frequency is used to drive the piezoelectric sensor, which results in electric current measurement difficultly for the sampling frequency restriction of the recording equipment. To measure high-frequency electric currents, a proposed measuring structure was assembled, as shown in Figure 14a. An external resistance connected in series with the cantilever piezoelectric sensor is proposed to obtain input current information from its cross voltage, where the current flows through a bridge rectifier circuit to transform it into DC current. Therefore, a general microchip controller, namely Arduino, can be utilized to recode the input current to estimate the impedance variation. Moreover, to achieve modularization, a general small-drive circuit for the piezoelectric transducer with 40 kHz is utilized in this paper, the electric impedance distribution of which is related with the external loading around the drive frequency, as measured in Figure 14b. A linear relation can be observed in that the system impedance is reduced in proportion to the external loading. Considering the drive behaviors of high voltages with small current compared with electrical motor, a large of series resistance is proposed to amplify its voltage drop. Therefore, a 10 kΩ series resistance was chosen, and its applied voltage is measured and recorded in Figure 15 with different standard weights. It can be seen that a loading force from the standard weights can result in the variable voltage drop across the same series resistance, and a fluctuation voltage in each measuring force can be found as around 0.2 Vpp. Moreover, the standard weights decreased the system impedance and resulted in higher measuring voltages at the series resistance, which corresponds with Figure 14b. Therefore, the linear proportional relation can be calculated by the curve-fitting method as follows:

(16) $$F_{s}=157V_{e}-214.5,$$

where $F_{s}$ is the loading force on the cantilever piezoelectric sensor, and $V_{e}$ is the voltage drop across the series resistance.

**Figure 16:**
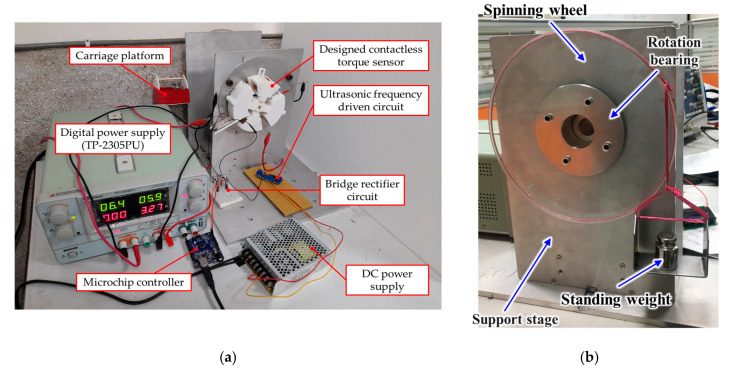
The performance measuring mechanism built to demonstrate the torque detection: (**a**) the designed contactless torque sensor is applied on the testing mechanism; (**b**) the testing structure of a known loading torque.

In order to demonstrate the measuring performance of the designed torque sensor, a testing mechanism for an external loading torque simulation was built, as shown in Figure 16. The inner ring of the designed contactless torque sensor is fixed on the support stage of the testing mechanism, and the outer ring is connected with the testing mechanism. By placing a standard weight on the spinning wheel, where the rotation radius is around 100 mm, a stable external torque loading can be simulated to test the designed torque sensor. To measure the repulsive force, the designed cantilever piezoelectric sensor is also mounted on the support stage, and its free side is coupled with the outer ring of the designed torque sensor. With an Arduino Card microchip controller, the voltage drop across the external resistance in series can be recoded and estimate the torque loading by passing through the bridge rectifier circuit. Finally, a digital DC power supply is utilized to drive the electromagnetic coil, and its field strength can be controlled by input current adjustment. Therefore, when a known stable torque loading is applied and adjusted by the standard weight, the external loading can be resisted by the strength of the electromagnetic coupling field, which depends on the input current of the coil. Then, the repulsive force can be estimated by measuring the voltage drop across the series resistance from the designed cantilever piezoelectric sensor.

**Figure 17:**
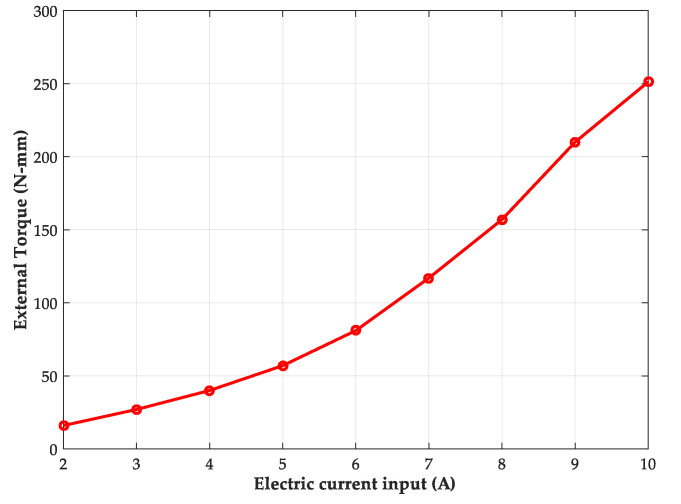
The measured data of the maximum measuring torque with different electric currents in the electromechanical coil.

Some standard weights, namely 30 g, 50 g, 80 g, and 130 g, were used to demonstrate the measurement accuracy of the designed contactless torque sensor. Including the weight of the carriage platform at around 75 g, the actual torque loading can be calculated and then compared with the estimation from the force–voltage relationship of Figure 15 according to the measured value of the voltage drop. The comparison results in Table 4 demonstrate the effectiveness of the proposed contactless torque sensor. Since the fluctuation occurs to the measuring voltage value, the estimated value of the loading torque also has oscillated. The maximum and minimum estimated torque is also recorded. To verify the correctness of the theoretical estimation, the average error comparing the actual loading with the estimated value is utilized, less than 10% of which can be found. Moreover, according to the prior theoretical analysis, the measuring range of the external torque loading can be adjusted by the electric current in the electromechanical coil of the designed contactless torque sensor. Figure 17 shows the measurement result of the maximum allowable torque loading depending on the electric current in the electromechanical coil. It can be found that the detecting range of the external torque can be adjusted from 7.8 to 125.6 N-mm when the drive current input ranges from 2 to 10 A. Moreover, the maximum allowable torque can be observed to increase sharply when a higher electric current is input in the coil. This increasing relationship is represented as a quadratic function related with the input current. Therefore, the achievement of an adjustable contactless torque sensor is demonstrated, where the torque measuring range is adjusted depending on the measuring requirement, and the measuring error is less than ±10%.

## 5. Discussion

This paper demonstrated a novel contactless torque sensor with notable thinness in its dimension, for which the external loading can be resisted by the strength of an electromagnetic coupling field depending on the input current of the coil. To observe the magnetic field strength related to the interval distance, the electromagnetic coil was proposed to be simulated as an equivalent magnetic piece, and finite element analysis software Ansoft was employed to simulate the coupling strength of two magnetic fields. The relation of the two coupling strengths with longitudinal direction is corresponded to the theoretical function of Equation (1). Nevertheless, for the proposed rotation structure, the strength in the radial direction should be generated to result in offset alignment, and a ball bearing was chosen to restrict in rotation movement. In addition, a cantilever piezoelectric sensor was designed to estimate the torque load. However, the voltage generated from the piezoelectric cantilever beam by the external force gradually decays with time by its inherent electric-loss property. Then, the external in-series resistance was proposed to measure its applied voltage, from which the electric impedance can be estimated to obtain the static loading force. In order to obtain the sufficient applied voltage, the appropriate resistance should be chosen to avoid the circuit broken in large electric impedance. In this paper, a 10 kΩ series resistance was proposed to demonstrate the 6% maximum estimated error from the compared results.

## 6. Conclusions

In this paper, a novel contactless torque sensor with notable thinness in its dimension by using an electromagnetic coil combined with permanent magnetic was proposed and realized, for which the external loading can be resisted by the strength of an electromagnetic coupling field depending on the input current of the coil. To design and analyze the sensor behaviors, a finite element analysis was employed to simulate the strength of the coupling-field intensity and show that it can be increased significantly when the induction coil is close to the permanent magnet or by increasing the input current of the electromagnetic coil. In addition, a cantilever piezoelectric sensor was designed to estimate the torque load value by measuring its electric impedance variation. According to the measured results, the proposed piezoelectric loading sensor demonstrated the accuracy of the proposed theoretical method, the error of which between the actual and estimated values was less than 10%. Finally, in order to realize the effectiveness of the theoretical analysis, a non-contact radial torque-sensing system with 11 cm in diameter and 2 cm in thickness was successfully designed, for which the detection ranges from 7.8 to 125.6 N-mm when the drive current input was from 2 to 10 A. Therefore, the designed contactless torque sensor can be used in intelligent automated technologies for its characteristics of thin dimensions, adjustable measuring range, and detection resolution.

## Figures and Tables

**Table 1:** Parameters of the designed electromagnet coil and chose permanent magnet.

Designed Electromagnet Coil		Chosen Permanent Magnet	
Parameter	Value	Parameter	Value
Material of round bar	Steel (304)	Material	NdFeB
Diameter of round bar	6 mm	Diameter	12 (mm)
Length of round bar	60 mm	Thickness	6 (mm)
Diameter of wire	0.7 mm	Magnetic flux density (Remanence, *B_r_*)	1.29 (Tesla)
Number of turns	300 turns	Coercivity	990 (kAm)
Relative magnet permeability	1–1.5 [14]	Density	7.49 (gcm3)
		Curie temperature	310 (°C)

**Table 2:** Dimensions and parameters of the proposed piezoelectric sensor with cantilever unimorph structure to measure the external force.

Piezoelectric Plate (PZT-5H)			Substrate Plate (SAE 304)		
Variable	Definition	Value	Variable	Definition	Value
$L_{P}$	Length	30 mm	*L_S_*	Total length	58 mm
*W_b_*	Width	15 mm	*W_b_*	Width	15 mm
*t_p_*	Thickness	0.3 mm	*t_s_*	Thickness	0.3 mm
*E_p_*	Young’s modulus	127.2×109 Nm2	*L_a_*	Distance from fixed end	200×109 Nm2
*d*_31_	Piezoelectric charge constant	−274×10−12 CN	*L_c_*	Distance from free end	5 mm
$\varepsilon_{33}^{T}$	Permittivity coefficient	3400$\varepsilon_{0}$	*E_s_*	Young’s modulus	23 mm
$\varepsilon_{0}$	Permittivity in vacuum	8.854×10−12 Fm			

**Table 3:** Designed dimensions of the proposed contactless torque sensor.

Dimensions	Value	Dimensions	Value
Diameter	110 mm	Diameter from center of magnet	90 mm
Thickness	20 mm	Range of interval distance	±10 mm
Number of poles	2	Electric current input	1–10 A

**Table 4:** Torque estimation comparison of the proposed contactless torque sensor.

Actual Torque $T_{A}$	Estimation Value $T_{E}$ (N-mm)			Average Error $\left(\left|\frac{T_{E}-T_{A}}{T_{A}}\times 100\%\right|\right)$
	Max	Min	Average	
103 N-mm	91 N-mm	101 N-mm	96 N-mm	6%
122.5 N-mm	134 N-mm	124 N-mm	129 N-mm	5.7%
152 N-mm	166 N-mm	153 N-mm	159 N-mm	2.6%
205 N-mm	205 N-mm	191 N-mm	198 N-mm	3.4%

## Data Availability

Not applicable.
